# Combined effect of nitrogen-doped functional groups and porosity of porous carbons on electrochemical performance of supercapacitors

**DOI:** 10.1038/s41598-021-97932-x

**Published:** 2021-09-15

**Authors:** Anna Ilnicka, Malgorzata Skorupska, Mariusz Szkoda, Zuzanna Zarach, Piotr Kamedulski, Wojciech Zielinski, Jerzy P. Lukaszewicz

**Affiliations:** 1 Faculty of Chemistry, Nicolaus Copernicus University in Torun, Gagarina 7, 87-100 Torun, Poland; 2 Department of Chemistry and Technology of Functional Materials, Faculty of Chemistry, Gdańsk University of Technology, Narutowicza 11/12, 80-233 Gdańsk, Poland; 3 Advanced Materials Center, Gdańsk University of Technology, Narutowicza 11/12, 80-233 Gdańsk, Poland; 4 Centre for Modern Interdisciplinary Technologies, Nicolaus Copernicus University in Torun, Wilenska 4, 87-100 Torun, Poland

**Keywords:** Electrochemistry, Materials chemistry, Surface chemistry

## Abstract

In this work, nitrogen-doped porous carbons obtained from chitosan, gelatine, and green algae were investigated in their role as supercapacitor electrodes. The effects of three factors on electrochemical performance have been studied—of the specific surface area, functional groups, and a porous structure. Varying nitrogen contents (from 5.46 to 10.08 wt.%) and specific surface areas (from 532 to 1095 m^2^ g^−1^) were obtained by modifying the carbon precursor and the carbonization temperature. Doping nitrogen into carbon at a level of 5.74–7.09 wt.% appears to be the optimum for obtaining high electrochemical capacitance. The obtained carbons exhibited high capacitance (231 F g^−1^ at 0.1 A g^−1^) and cycle durability in a 0.2 mol L^−1^ K_2_SO_4_ electrolyte. Capacitance retention was equal to 91% at 5 A g^−1^ after 10,000 chronopotentiometry cycles. An analysis of electrochemical behaviour reveals the influence that nitrogen functional groups have on pseudocapacitance. While quaternary-N and pyrrolic-N nitrogen groups have an enhancing effect, due to the presence of a positive charge and thus improved electron transfer at high current loads, the most important functional group affecting energy storage performance is graphite-N/quaternary-N. The study points out that the search for the most favourable organic precursors is as important as the process of converting precursors to carbon-based electrode materials.

## Introduction

Tremendous attention is currently being paid to a variety of porous materials^1–4^, metal organic frameworks^5^, and metal oxides^6,7^ in the context of their application as electrode materials in supercapacitors due to their high electric conductivity and ability to operate in various electrolytes^8–10^. Similarly, it is evident that well-designed hierarchically porous carbons^11–13^, graphene quantum dots^14^, three-dimensional graphene foams^15^, or porous carbons with interconnected pores play a crucial role in ion transport^16,17^.

Supercapacitors whose pseudocapacitance originates from heteroatoms or functional groups on the electrode–electrolyte interface are being widely investigated and appear very promising^18^. Functionalization of carbon materials with heteroatoms such as sulphur^19,20^, boron^21,22^, phosphorus^23–25^, nitrogen^26–28^, and oxygen^29,30^ was proven to have a significant influence on the improvement of supercapacitor performance^16,31^. Nitrogen and oxygen functionalities can increase the wettability, electrical conductivity, and the contribution of pseudocapacitance^32–34^. Aside from surface chemistry, the porosity of carbon electrodes also has a considerable effect on the value of capacitance^35–37^. The electrode materials of choice are carbon materials containing an adjustable pore structure and surface features which are favourable for electrolyte ion storage and electron/ion transfer^38–40^. Carbons obtained from biomass are particularly full of potential due to their elemental composition and low cost^20,41–43^. Those derived carbons from corncob lignin have a high rate of performance in electrolytes with high voltage ranges, such as LiCl and Li_2_SO_4_^44^. In the case of using activated carbons as electrode materials, a stable potential window of an electrolyte is crucial. Tang et al. demonstrated that a quantitative analysis of carbon edge sites by means of high-temperature programmed desorption, up to 1800 °C, is an effective tool to judge the electrochemical stability of carbon materials and understand the corrosion reaction mechanism^45^. In a paper by Nomura et al., edge-free graphene walls were found to cause ultra-high stability at 4.4 V of a supercapacitor with organic electrolyte at 25 °C^46^. Research by Tang et al. also confirmed that the initial degradation reactions mainly occurred at carbon basal planes rather than edge sites^47^.

Neutral electrolytes are more environmentally friendly compared to acidic and alkaline ones and their large stable potential window^48^ gives them a lot of promise. Potassium (e.g., KCl, K_2_SO4, and KNO_3_) salts are the preferred conducting salts to use as electrolytes in supercapacitors containing carbon electrodes^49^. For nitrogen-doped carbon electrodes, the contribution of capacitance in K_2_SO_4_ electrolyte by nitrogen groups is found to be higher at low current densities; this is because of the strong adsorption of K^+^ to pyrrole-like nitrogen configurations^50^. It therefore follows that K_2_SO_4_ is an electrolyte very likely to be successful with many pseudocapacitive materials (especially N-doped materials). According to the literature, nitrogen-doped carbon materials have already been obtained from sources like gelatine^51,52^, algae^53^, and chitosan^54–56^. However, as far as the authors are concerned, these carbon structures have not yet been adequately studied in a K_2_SO_4_ electrolyte.

As pointed out above, N-doped activated carbons offer a breadth of opportunities to improve the electrochemical performance of supercapacitors; the authors took advantage of this and simultaneously performed N-doping and tailored the pore structure in the present study. The research concept is based on their previous experience in N-doping using organic matter as a precursor for carbon electrode manufacturing. To achieve the aim of this paper, the possible controlling influence on N-doping of three kinds of carbon precursors was investigated, namely that of green algae, chitosan, and gelatine. Characterization of carbon materials was performed through an examination of surface morphology, elemental composition, and chemical structure, so that their pore structure could be tailored and classified by means of low temperature gas adsorption. The complex characteristics of the surface and elemental species were satisfactory for electrochemical application. In our previous paper, we reported the materials mentioned above as catalysts for the oxygen reduction reaction^57^. In this research, those materials structures and properties were evaluated that had accounted for the promising supercapacitors performance.

## Results and discussion

### Materials characterisation

**Figure 1:**
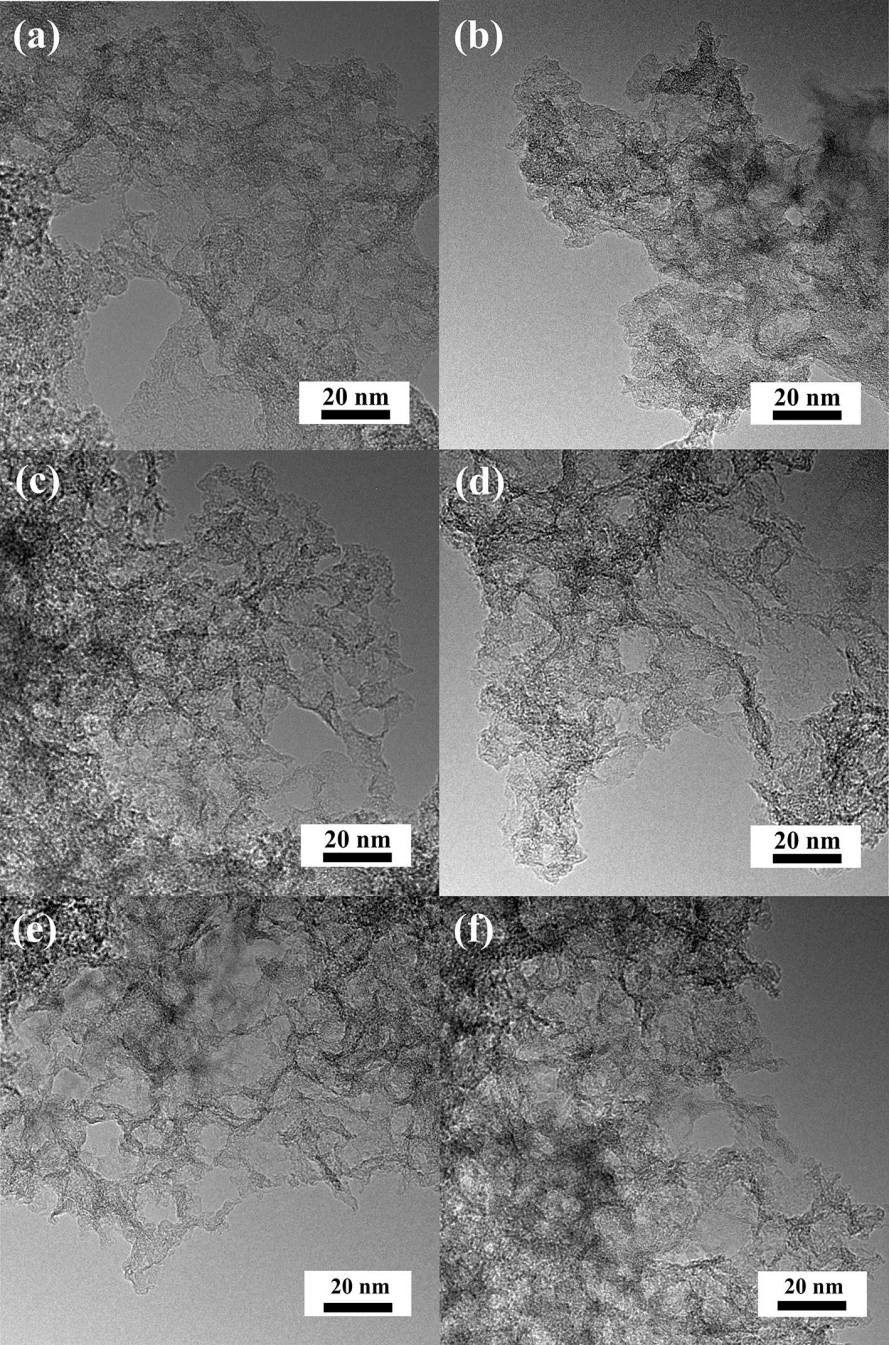
HRTEM images of (**a**) N-APC-800, (**b**) N-APC-900, (**c**) N-GPC-800, (**d**) N-GPC-900, (**e**) N-CPC-800, (**f**) N-CPC-900.

The morphology and microstructure of porous materials was characterized via high-resolution transmission electron microscopy (HRTEM). HRTEM images (Fig. 1) of samples synthesized from chitosan (N-CPC-800 and N-CPC-900) present a uniform and similar network structure to samples obtained from gelatine (N-GPC-800 and N-GPC-900) and algae (N-APC-800 and N-APC-900), as demonstrated in our previous paper^57^. A porous structure can contribute to the diffusion and transfer of ions from the bulk solution to the material’s inner-surface.

**Figure 2:**
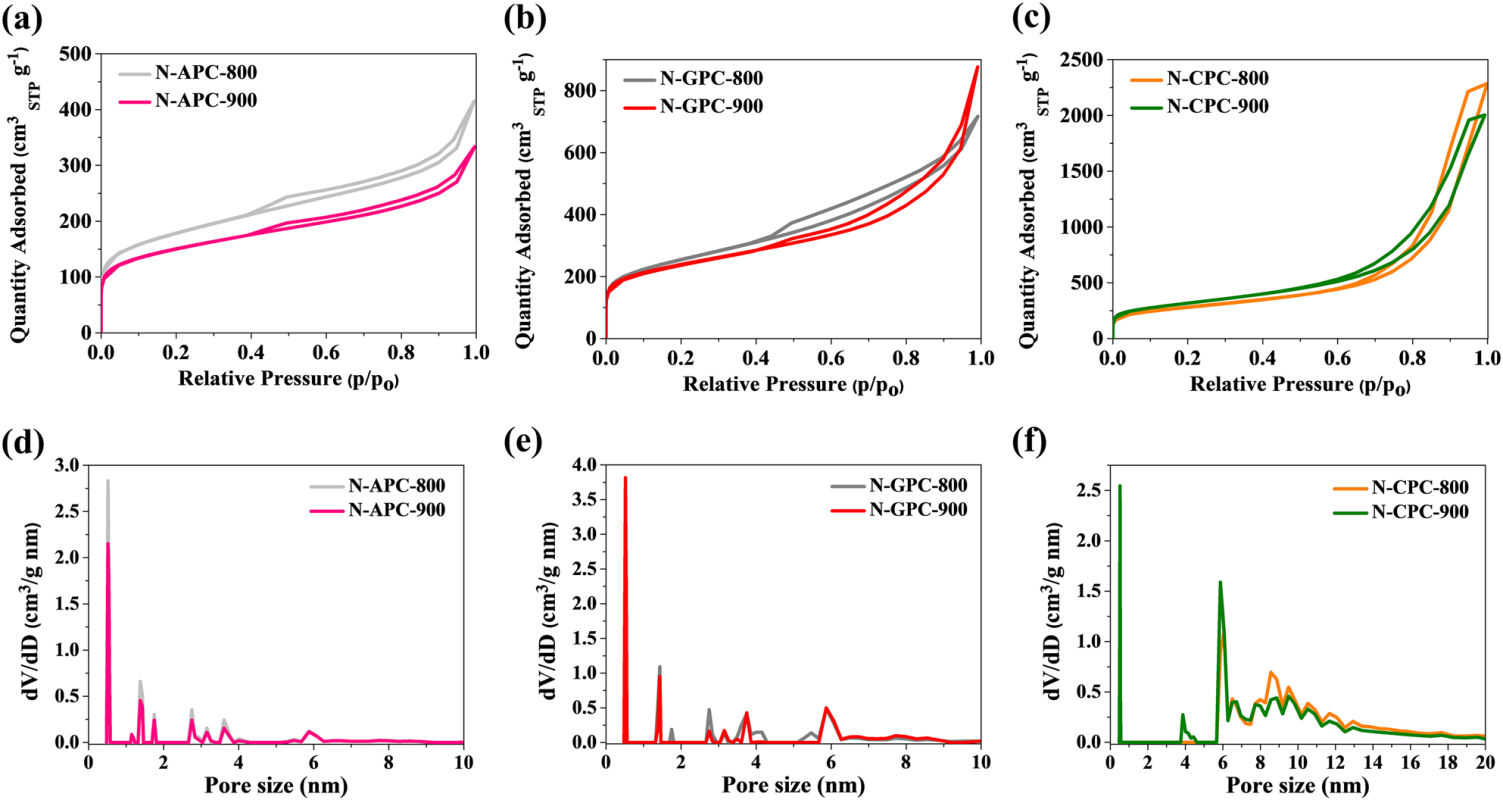
(**a**–**c**) Nitrogen adsorption–desorption isotherms, (**d**–**f**) pore size distribution calculated from N_2_ sorption isotherms using the DFT method.

Samples were analysed by means of N_2_ adsorption measurements at − 196 °C in order to acquire more detailed textural features of the different porous carbons at different activation temperatures (800 and 900 °C). The adsorption–desorption isotherms presented in Fig. 2a–c visibly demonstrate the combination of type-I and type-IV adsorption isotherms, characteristic of porous carbon materials, according to the IUPAC classification^58^. The textural parameters gained from nitrogen adsorption–desorption data are additionally listed in Table 1. From there it is visible that the temperature of thermal treatment can affect the porous structure, as can the carbon precursor. Sample N-CPC-900 had the highest specific surface area at 1095 m^2^ g^−1^. Carbons prepared at 800 °C exhibited higher values of S_mi_/S_total_ for algae- and gelatine-derived samples and a lower value for the chitosan-derived sample. Pore size distribution (PSD) was calculated using density functional theory (DFT), which assumes a slit geometry for micropores and a cylindrical pore geometry for mesopores. The presented PSD reveals the existence of well-defined micro- and meso-pores with sizes of less than 20 nm (Fig. 2d-f). The highest specific surface area for algae- and gelatine-derived samples was 623 m^2^ g^−1^ and 880 m^2^ g^−1^, respectively. The specific surface area for carbons obtained from a chitosan precursor increased from 972 to 1095 m^2^ g^−1^ when the carbonization temperature was raised, while the pore volume decreased from 3.65 to 3.22 cm^3^ g^−1^. A general tendency in gelatine and algae precursors is clearly visible: when carbonization temperature increases from 800 to 900 °C, the specific surface area decreases, as previously reported^57^. The samples’ elemental compositions showed nitrogen levels between 5.74 and 10.08 wt.%, indicating that a remarkably high amount of nitrogen remained bound in the final structures after high-temperature carbonization.

**Table 1:** Physicochemical properties and elemental composition via bulk combustion of carbon samples.

Carbon sample	Elemental content (wt.%)			S_BET_^a^ (m^2^ g^−1^)	S_mi_^b^ (m^2^ g^−1^)	S_mi_/S_BET_ (%)	V_total_^c^ (cm^3^ g^−1^)	V_micro_^d^ (cm^3^ g^−1^)	V_meso_^e^ (cm^3^ g^−1^)	V_micro_/V_total_ (%)	V_meso_/V_total_ (%)
	C	H	N								
N-GPC-800	68.28	2.19	10.08	880	692	78.6	1.12	0.38	0.74	33.9	66.1
N-GPC-900	73.89	1.09	7.41	842	577	68.5	1.39	0.36	1.03	25.9	83.1
N-APC-800	65.60	2.01	7.09	623	446	71.6	0.64	0.27	0.37	42.2	57.8
N-APC-900	64.92	1.88	5.74	532	346	65.0	0.51	0.23	0.28	45.1	54.9
N-CPC-800	80.19	1.56	8.32	972	809	83.2	3.65	0.43	3.22	11.8	88.2
N-CPC-900	87.15	1.24	5.46	1095	946	86.4	3.22	0.48	2.74	14.9	85.1

^a^Specific surface areas were obtained through the Brunauer–Emmett–Teller (BET) method.

^b^Micropore surface was area acquired by means of the t–plot method.

^c^Total pore volume was calculated using the Density Functional Theory (DFT) method.

^d^Micropore volume was measured using the Horvath-Kawazoe method.

^e^Mesopore volume was calculated by subtracting V_micro_ from V_total_.

**Figure 3:**
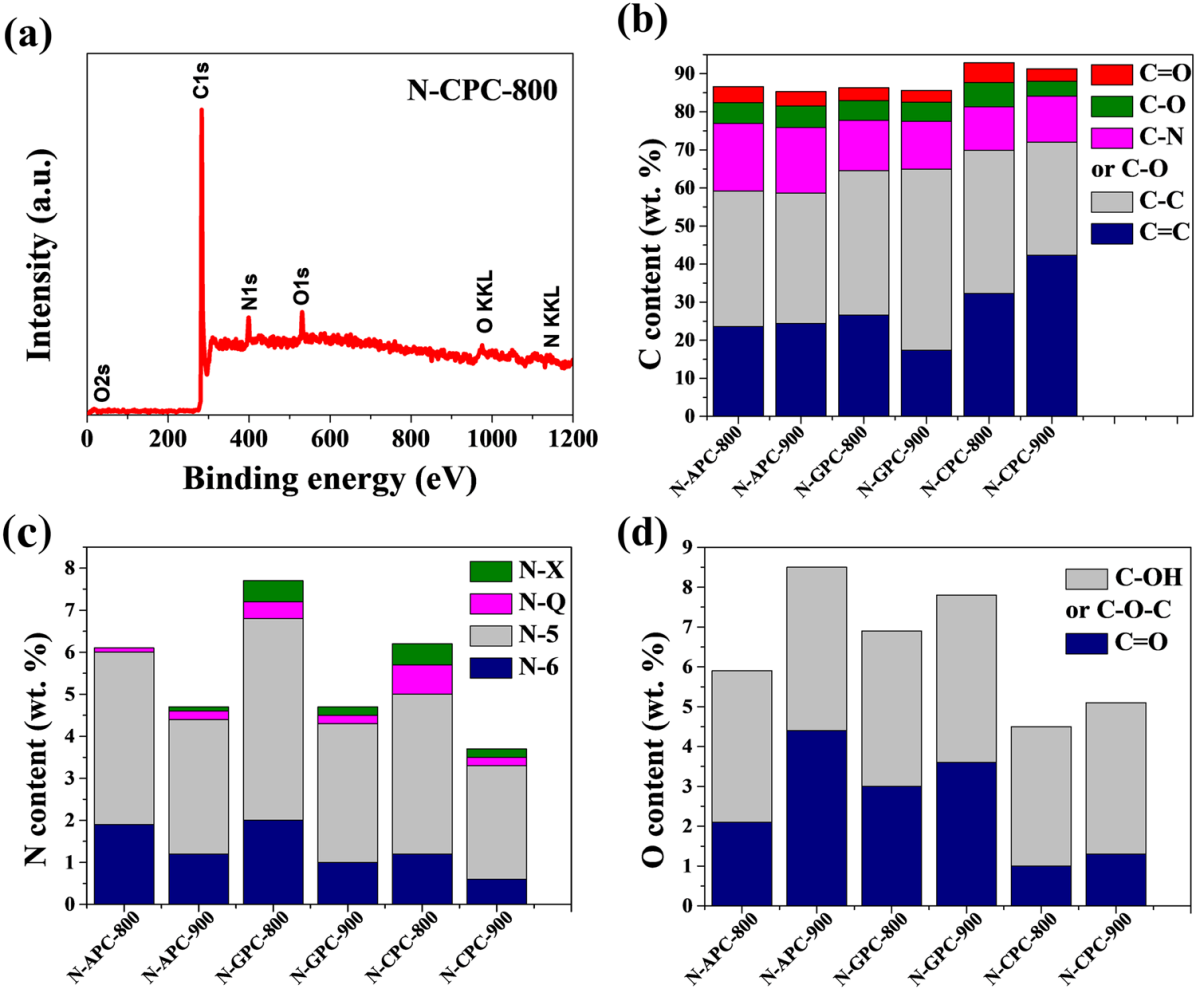
(**a**) XPS survey spectrum taken from the surface of N-CPC-800 sample. Content percentages of different (**b**) carbon species, (**c**) nitrogen species, (**d**) oxygen species.

XPS survey was employed to analyse the surface chemical properties for all as-prepared carbon materials. These properties were found to be affected by the type of carbon precursor and the specific groups present on its surface before carbonization. As presented by Fig. 3a, the survey spectrum for N-CPC-800, the material possessed the same elements as other samples and exhibited three peaks corresponding to C1s, N1s, and O1s, which is a confirmation of heteroatoms (N, O) doping into the carbon matrix during the carbonization process. The elemental composition, presented in Fig. 3 b-d, was retrieved from the high resolution of XPS spectra. The high resolution of C1s XPS spectra consisted of five peaks located at 284.6 eV, 285.0 eV, 286.4 eV, 287.7 eV, and 288.6 eV, which refer to C=C, C–C, C–N or C–O, C–O, C=O, respectively^12,20,38^. The high resolution of N1s XPS spectra consisted of four types of bonding, identified as pyridinic N (N-6), pyrrolic N (N-5) and graphitic N (N-Q) and pyridinic N oxide (N-X) at 398.3 eV, 400.4 eV, 402.5 eV and 404.5 eV, respectively^13,18,33,39^. Recently, research has plainly stated that N-5 can provide available chemical active sites for the faradaic reaction, resulting in significant pseudocapacitance^59–61^. The presence of nitrogen atoms and a well-developed surface area have a significant impact on providing pseudocapacitance and enhancing the capability for charge accumulation in the electric double layer formed at the electrode/electrolyte interface. N-Q can also contribute to electron transport^37,62^. In the fitting of O1s spectra, all samples that revealed two peaks at 531.3 eV and 533.2 eV were assigned to the C=O carbonyl- or quinone-type groups and C–OH phenol or C–O–C ether groups, respectively^18,42,63^.

### Electrochemical performance

#### Three-electrode configuration

**Figure 4:**
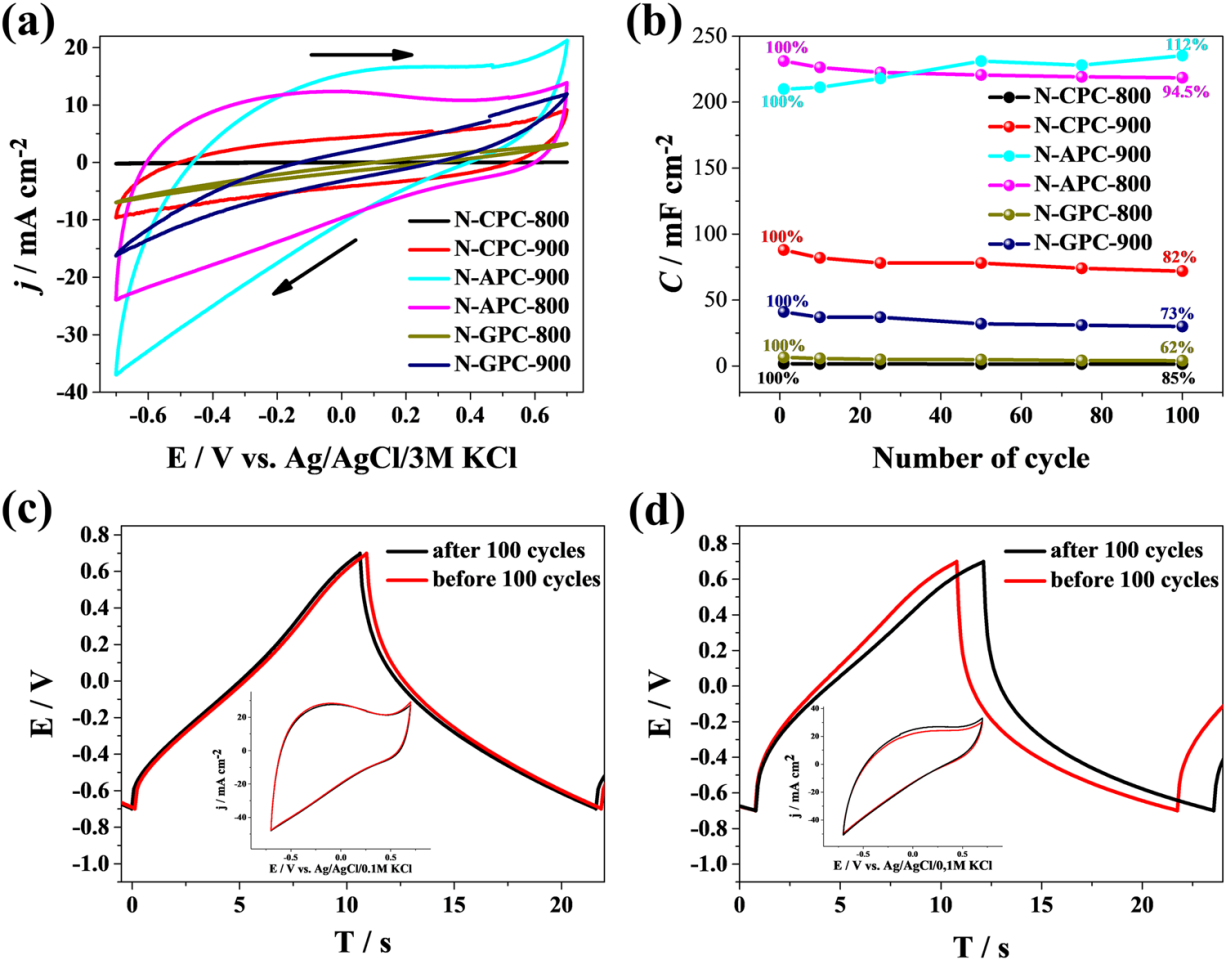
(**a**) Cyclic voltammetry curves recorded for N-doped carbon materials in 0.2 mol L^−1^ K_2_SO_4_ with a potential window between − 0.7 V and + 0.7 V (*v* = 50 mV s^−1^). (**b**) Specific capacitance plotted as a function of the number of cycles for investigated carbon materials. Exemplary galvanostatic charge–discharge curves for N-APC-800 and (**d**) N-APC-900, recorded at 5 A g^−1^ (inset: cyclic voltammetry curves recorded in 0.2 mol L^−1^ K_2_SO_4_ (*v* = 50 mV s^−1^)).

In order to verify the effectiveness of N-doped porous carbons as electrode materials for supercapacitors, several electrochemical measurements were carried out, including cyclic voltammetry (CV) and galvanostatic charge–discharge (GDC). A three-electrode system was used, consisting of Ag/AgCl/3 M KCl as the reference electrode, N-doped carbon as the working electrode, and Pt mesh as the counter electrode. The electrochemical measurements were performed in 0.2 mol L^−1^ K_2_SO_4_ as a neutral aqueous electrolyte. CV measurements were taken for a number of electrodes: N-APC-800, N-APC-900, N-GPC-800, N-GPC-900, N-CPC-800, and N-CPC-900 (Fig. 4a); each measurement was performed in a voltage window of − 0.7 to + 0.7 V. As shown in Fig. 4a, the curves of N-GPC-800, N-GPC-900, N-CPC-800, and N-CPC-900 exhibit an irregular shape; the values of generated currents indicate unsatisfactory supercapacitive behaviour. The CV curves of two other samples (N-APC-800 and N-APC-900) exhibit an approximately rectangular shape, which indicates the co-contribution of both electric double layer capacitance (EDLC) and a significantly reversible Faraday effect, mainly caused by the presence of heteroatoms or functional groups on the carbon materials’ surface^47^.

By carrying out a more detailed analysis of the obtained results, it was possible to note a significant correlation regarding nitrogen content in the electrode material (see Table 1 and Fig. 4b)—materials with the highest nitrogen content are characterized by the lowest specific capacitance values. Moreover, it is evident that the nitrogen content should be kept within an appropriate range, as values greater than 7.09% or lower than 5.74% correspond to a drop of specific capacitance, especially when the doping content is increased^64^. In general, the literature demonstrates that an increase of nitrogen doping causes an increase of the specific capacitance value^65–67^. However, it should be remembered that the overall performance of electrode materials is additionally influenced by other parameters that may be closely related to each other and have a synergistic effect on capacitance values. As a result, the contribution of particular types of pores (micro- and mesopores) in their total volume may be a crucial parameter as well. Several recent studies show that the pore size, in particular, is critical for the improved performance of carbons in supercapacitor applications^68,69^. Analysing the contribution of these factors, materials with a similar share of the two pore types, namely N-APC-800 and N-APC-900, are characterized by the highest capacitance. Mesopores serve as ion highways, which enable fast ion transport into the bulk of the material, and therefore contribute to a high power density^70^. They can also function as a host for pseudocapacitive species and eventually lead to enhanced capacitance linked with fast faradaic reactions, thus increasing the energy performance of a device^71^. Furthermore, N-APC-800 and N-APC-900 electrode materials possess the highest number of micropores, which is of great benefit to its charge storage capability and rate performance^72^. Considering pore type and nitrogen content, it was also demonstrated that nitrogen doping may cause a decrease in micropore volume through the destruction of pore walls and micropore blocking by functional groups^66,67^. Therefore, it seems that both properly balanced nitrogen content and similar contributions of micro- and meso-pores augment overall electrochemical performance. A surprising observation may be the fact that the highest capacitance is obtained for materials with the smallest specific surface area. Hence, the gain in specific capacitance does not seem to be directly related to an increase of S_BET_. It is worth mentioning that the race for the highest surface area is no longer as beneficial as it once was, and it has dwindled as the maximum theoretical limit appears to have been achieved^73^.

Multiple galvanostatic charge–discharge (GCD) curves were obtained from measurements carried out in 0.2 mol L^−1^ K_2_SO_4_ in a potential range from − 0.7 to + 0.7 V with 30 mA cm^−2^ current applied. As shown in Fig. 4c,d, the capacitance curve profiles displayed what were almost isosceles triangles with a small distortion, confirming the reversible accumulation of ions and a pseudocapacitance effect in the redox reaction. The resulting faradaic pseudocapacitance is attributed to oxygen (hydroxyl and quinone) and nitrogen (pyridinic and pyrrolic groups) functionalities^42^. The N-APC-800 electrode material showed capacity retention of about 95% after 100 cycles, which is entirely confirmed by the results of cyclic voltammetry (see the insets of Fig. 4c,d), as in both cases the areas under the have an almost identical value. Conversely, for the N-APC-900 electrode material there was a slight increase in overall capacitance value, and thus an increase of the capacitance retention value (112%) could be observed.

**Figure 5:**
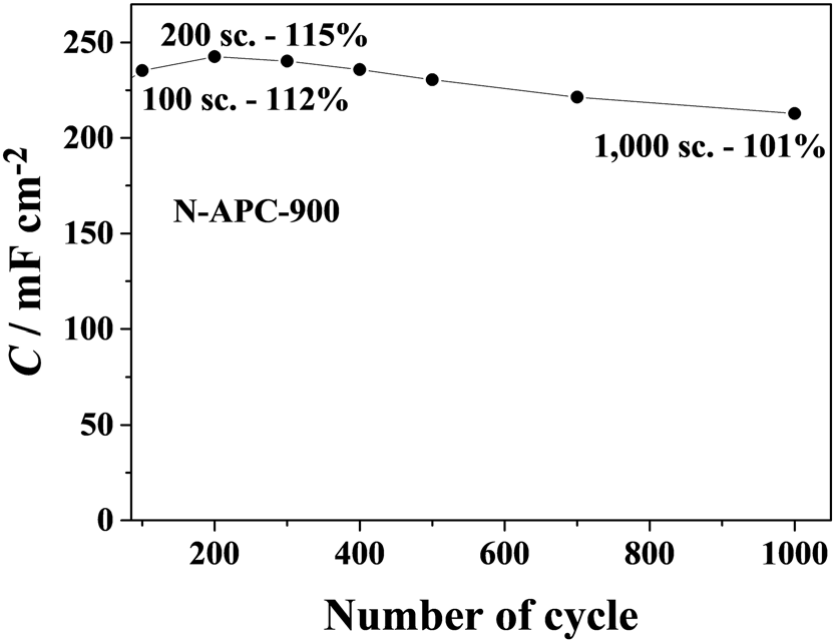
Curves of specific capacitance plotted as a function of cycle number for N-APC-900 (1000 cycles).

In order to investigate whether the phenomenon is repeatable and the capacitance value increases over a longer period, measurements over 1,000 cycles were performed. The results are presented in Fig. 5. An increase in capacitance value was observed for the first 200 cycles, after which capacitance began decreasing. However, after 1,000 cycles the level was still outstanding and slightly higher than at the beginning (101%). The capacitance increase may be related to carbon surface activation and the formation of additional surface groups caused by electrode polarization. The observed phenomenon may also be the consequence of pore size. A large number of micropores makes it difficult for the electrolyte to diffuse deep into the porous carbon and therefore, after some time, a maximum value of capacitance is reached.

#### Two-electrode configuration

**Figure 6:**
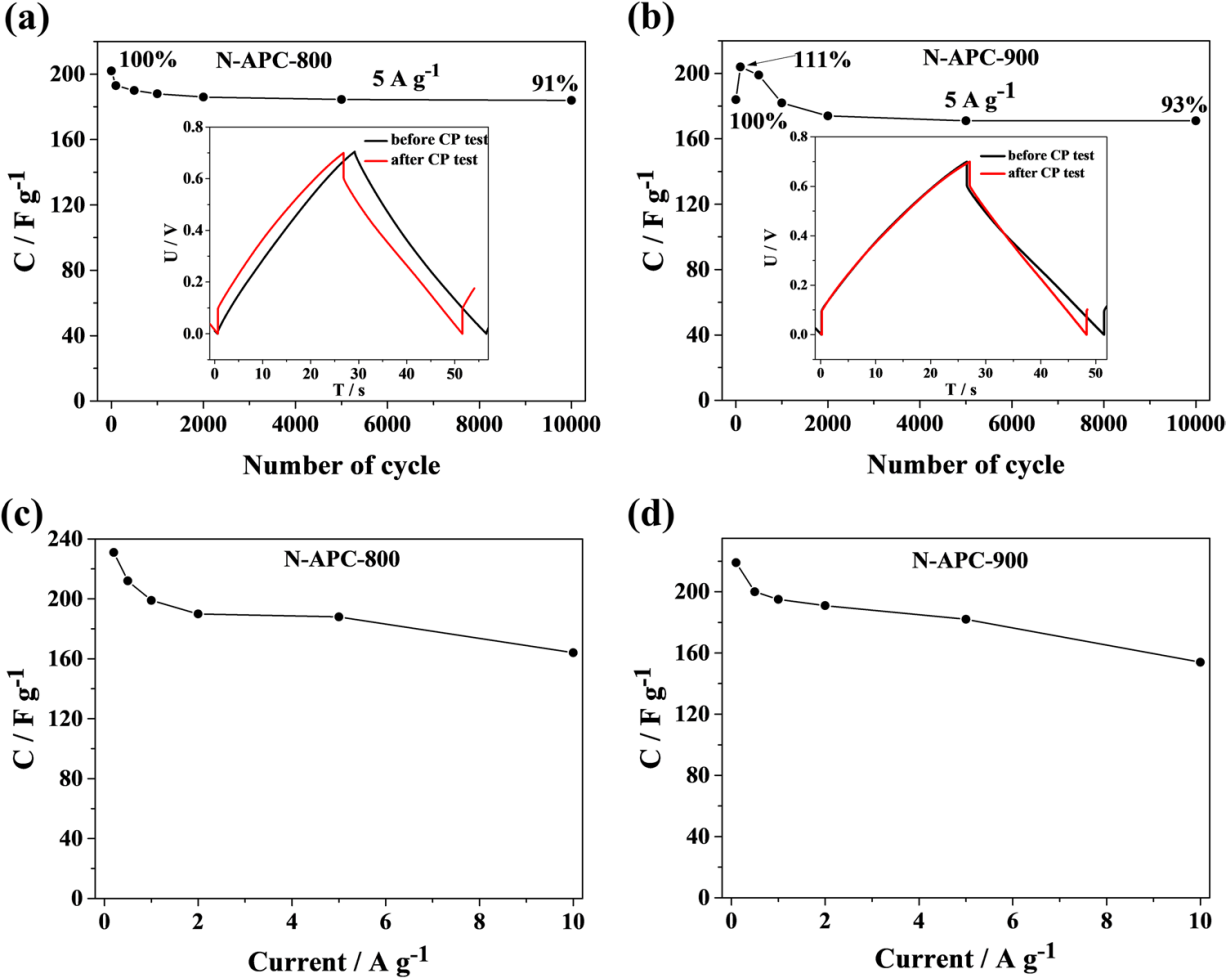
Curves of specific capacitance plotted as a function of cycle number for (**a**) N-APC-800 and (**b**) N-APC-900. Insets: exemplary galvanostatic charge–discharge curves for the electrode materials recorded at 5 A g^−1^. Specific capacity as a function of the current density applied in charge–discharge measurements for (**c**) N-APC-800 and (**d**) N-APC-900 symmetric supercapacitors.

Multiple charge–discharge cycles were performed in a two-electrode configuration for N-APC-800 and N-APC-900 in order to examine the stability of symmetric supercapacitors (Fig. 6) made for each of the carbon materials. Figure 6a,b exhibit GCD curves recorded at a current density of 5 A g^−1^ for N-APC-800 and N-APC-900, respectively. The specific capacitance (C_s_) values for both materials (after 2,000 cycles) are nearly indistinguishable at 186 F g^−1^ (N-APC-800) and 174 F g^−1^ (N-APC-900). All curves exhibit triangular shapes, indicating that porous N-doping carbon materials possess good electrochemical reversibility and behaviour characteristic of supercapacitors (Fig. 6a,b insets). As is possible to observe, stability above 90% was obtained for both supercapacitors, even after 10,000 cycles. In the case of N-APC-800, capacitance retention was equal to 91% up to the 10,000^th^ chronopotentiometry cycle.

The decrease of capacitance is the greatest at the beginning of GCD tests, but begins to stabilize after the 1000th cycle. A distinctly different behaviour can be observed for N-APC-900; a continuous increase in capacitance was observed between the 1st and 100th scan, after which it reached a maximum value of 204 F g^−1^. Later, the capacity value fell and eventually stabilized around the 2000th cycle. Specific capacitance values calculated from the charge–discharge curves recorded at different current densities are shown in Fig. 6c and 6d. The specific capacitance value for N-APC-800 at 0.1 A g^−1^ sits at around 231 F g^−1^ and remains fairly high, circa 164 F g^−1^, even with the discharge current density as high as 10 A g^−1^. Similarly, for the N-APC-900-based supercapacitor, good performance was dependant on the discharge current, as demonstrated by the C_s_ value being equal to 219 A g^−1^ and 154 A g^−1^ at a current density of 0.1 A g^−1^ and 10 A g^−1^, respectively.

**Figure 7:**
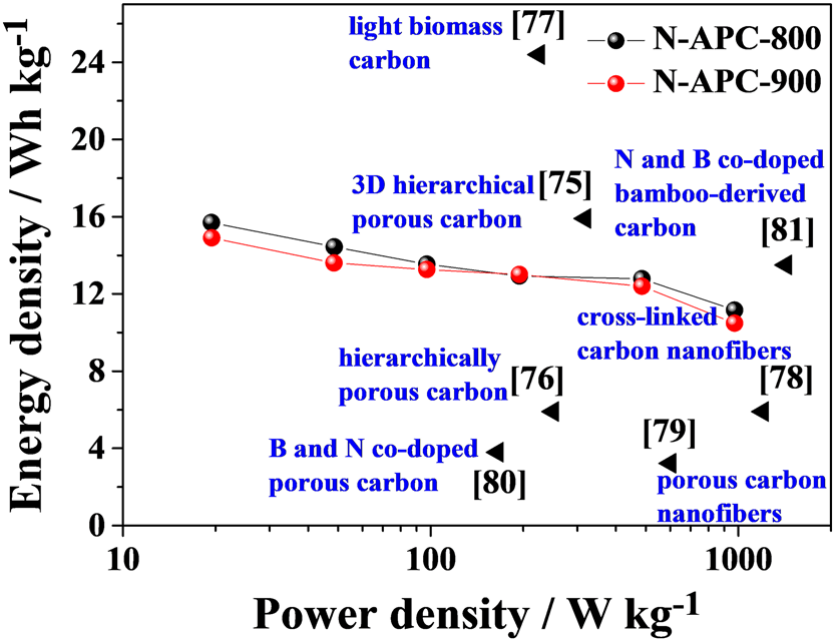
Ragone plots of N-APC-800 and N-APC-800 symmetric supercapacitors compared to other carbon-based supercapacitors.

As presented in Fig. 7, a Ragone plot for the N-APC-800 and N-APC-900 two-electrode device illustrates the relationship between energy density and power density obtained for different charge–discharge current densities (0.1, 0.5, 1, 2, 5, 10 A g^−1^). Energy density and power density using were calculated Eqs. (1) and (2), respectively:

1 $$E_{cell} = \frac{{1000 \cdot 1/2\left( {C_{cell} \cdot V^{2} } \right)}}{3600}$$

2 $$P_{cell} = \left( {\frac{{E_{cell} }}{t}} \right) \cdot 1000$$

where *E*_*cell*_ is the energy density based on the mass of the electrodes, *C*_*cell*_ is the electrode’s mass-based specific capacitance, *V* is the voltage charge during the discharge process, *P*_*cell*_ is the power density, and *t* is discharge time.

As is visible, the power and energy densities for both supercapacitors are comparable. In the case of N-APC-800, energy density of the symmetric supercapacitor equals 12.8 Wh kg^−1^ and its corresponding power density equals 486 W kg^−1^ (at a current density of 5 A g^−1^), whereas for N-APC-900, at a power density of 487 W kg^−1^, energy density of 12.4 Wh kg^−1^ was produced (at the same discharge current density). The obtained results are evidently higher than or comparable with earlier reports regarding porous carbon materials in supercapacitors^74–81^.

## Materials and methods

### Carbon preparation

The fabrication process of gelatine- and algae-derived porous carbons was described in a previous report^57^. The nitrogen-rich porous carbon materials were prepared using a solution of colloidal silica (SiO_2_), as well as green algae or gelatine at a weight ratio of 2.5:1. In the case of gelatine, an ammonium buffer was additionally used so that the solution’s pH remained in the range of 8.7–9.0. The resulting mass was stirred continuously until the deionized water evaporated, and was then carbonized at 800 or 900 °C under N_2_ flow. After carbonization, N-rich carbon was retrieved via removal of the silicate template using a hydrofluoric acid (HF) solution. It was repeatedly washed with deionized water, then dried at 120 °C for 24 h. Samples were labelled N-APC-800, N-APC-900, N-GPC-800, and N-GPC-900, where: N-APC-N-rich denotes porous carbon obtained from green algae, while N-GPC-N-rich denotes porous carbon obtained from gelatine. A carbonization temperature of 800 or 900 °C was denoted in the names of samples as 800 or 900, respectively.

In this paper, we also present new samples obtained from chitosan that haven’t been described previously. In the case of chitosan, 1 g of polymer was dissolved in 200 ml of a 1% acetic acid water solution at 80 °C while being constantly stirred. An water-suspended silica was added to the precursors in a 1:1.5 weight ratio of carbon phase precursor to SiO_2_. The samples were left on a magnetic stirrer until the water evaporated completely. Dried solid samples were carbonized in a tube furnace by heating them to 800 or 900 °C in an atmosphere of high-purity nitrogen, with a heating rate of 3 °C min^−1^. The samples were held at 800 or 900 °C for 1 h and eventually cooled to room temperature under N_2_ flow. Finally, SiO_2_ was removed through a 15% hydrofluoric acid treatment. Afterwards, the samples were washed on a Buchner funnel with distilled water until the pH of the effluent was neutral. The samples were dried at 120 °C overnight. Samples obtained from chitosan at 800 and 900 °C were denoted as N-CPC-800 and N-CPC-900, respectively.

### Chemical characterization

The carbons’ morphology was characterized using high-resolution transmission electron microscopy. The sorption of nitrogen was carried out using ASAP2020 Plus (Micromeritics). Before examination, samples were outgassed in a vacuum at 200 °C for 24 h. The specific surface area (S_BET_) was calculated using the Brunauer–Emmett–Teller (BET) method. The total pore volume (V_total_) and pore size distribution was calculated using the Density Functional Theory (DFT) method. The carbon, nitrogen, and hydrogen contents were measured by a bulk combustion analysis. X-ray photoelectron spectroscopy measurements were performed with a monochromatic Al Kα excitation source operated at 1486.6 eV. The survey and high-resolution spectra were collected with 0.5 eV and 0.1 eV pass energy, respectively. The spectra were taken after being referenced to the C1s neutral carbon peak at 284.8 eV.

### Electrochemical measurements

To investigate the electrochemical properties of the samples, both a three-electrode and a two-electrode configuration were used. In a three-electrode configuration, the electrode material was prepared using porous carbon, poly-vinylidenefluoride (PVDF), and acetylene black (porous carbon/acetylene black/PVDF weight ratio of 8:1:1). After stirring for 12 h, the mixture was dropped onto glassy carbon with a diameter of 1.5 mm and the prepared electrodes were dried at 60 °C. In a conventional three-electrode system, a Pt mesh and Ag/AgCl/3 M KCl were used as the counter electrode and the reference electrode, respectively. An aqueous solution of 0.2 mol L^−1^ K_2_SO_4_ was used as the electrolyte. A symmetric supercapacitor was also constructed by combining two GF (flexible graphite foil), obtained material electrodes and placing a fiberglass separator soaked in a 0.2 mol L^−1^ K_2_SO_4_ aqueous electrolyte between them (mixture was dropped on GF and dried at 40 °C for 6 h). The mass loading of the carbon materials (N-APC-800 and N-APC-900) was measured using the weight difference of the electrode material before and after dropping a mixture containing the tested carbon on GF; Analytical Balance RADWAG XA 82/220.4Y PLUS with an accuracy of 0.01 mg was employed. The mass equaled 5.12 and 6.26 mg for N-APC-800 and N-APC-900, respectively. In the next step, the casing foil was welded on three sides using a plastic foil welder, and finally the setup was sealed using a vacuum packing machine (CAS CVP-350/MS, Hertogenbosch, The Netherlands).

Cyclic voltammetric (CV) and galvanostatic charge–discharge measurements (GCD) were performed using BioLogic VSP 2078. In the three-electrode electrochemical cell, GCD measurements were carried out with a 30 mA cm^−2^ current density in a polarization range of -0.7 to 0.7 V. For all measurements, the electrolyte was initially purged with argon for 30 min in order to remove oxygen. The experiments were additionally carried out under argon atmosphere. For the symmetric supercapacitor, galvanostatic charge and discharge tests (10,000 cycles) were performed. Charge and discharge measurements were made with current density values in the range of 0.1 to 10 A g^−1^, in the electrochemical voltage range of 0 to 0.7 V.

## Conclusions

An investigation was undertaken to understand the influence that both nitrogen surface functional groups and the porous structure of carbons have on the capacitance of supercapacitors. For this purpose, a comprehensive surface characterization of carbons, an analysis of their porous structure, and electrochemical testing in two- and three-electrode cells in 0.2 mol L^−1^ K_2_SO_4_ were carried out. Quaternary-N and pyrrolic-N were shown to affect capacitance due to their positive charge and subsequent improved electron transfer. This was particularly the case at higher current loads, when double-layer capacitance is less pronounced than pseudocapacitance. However, subtle differences exist when considering nitrogen content in the precursor, chemistries of the precursors, and the reaction of the surface when carbons are exposed to heat treatment. These factors influence the porosity of carbons and result in different special distributions of groups on the surface. The charge on nitrogen atoms positively affects stability of the whole system, in particular the stability of the carbon itself, and the stability of the electrolyte. The N-doped carbon N-APC-800 exhibited good performance of N-doped carbon, reaching 91% capacitance retention at 5 A g^−1^. This mechanism is even more pronounced at higher current loads. Moreover, the as-assembled symmetric cell N-APC-800 sample displayed an energy density of 12.8 Wh kg^−1^ with a power density of 486 W kg^−1^ at a current density of 5 A g^−1^, which was higher than the N-APC-900 sample. Our primary assumption, that the electrochemical performance of bio-originated electrode materials is governed by two factors, i.e., N-content and porosity/surface, has only been partially confirmed. The results reveal that other factors related to the origin of the organic precursor (algae, chitosan, and gelatine) play a crucial role. All investigated samples had a high nitrogen content, well-developed pore structure/surface area, and were manufactured in a similar way. However, only APC-series carbon exhibits outstanding electrochemical parameters when tested as a supercapacitor electrode material. Thus, the study indicates that the search for optimal organic precursors is as important as the process of converting precursors to carbon-based electrode materials.
